# Changes of the Alveolar Bone Ridge Using Bone Mineral Grafts and Collagen Membranes after Tooth Extraction: A Systematic Review and Meta-Analysis

**DOI:** 10.3390/bioengineering11060565

**Published:** 2024-06-03

**Authors:** Nansi López-Valverde, Bruno Macedo de Sousa, José Antonio Blanco Rueda

**Affiliations:** 1 Department of Surgery, Biomedical Research Institute of Salamanca (IBSAL), University of Salamanca, 37008 Salamanca, Spain; jablancor@usal.es; 2 Institute for Occlusion and Orofacial Pain, Faculty of Medicine, University of Coimbra, 3004-531 Coimbra, Portugal; bsousa@fmed.uc.pt

**Keywords:** socket preservation, bone regeneration, bone mineral graft, collagen membrane, randomized clinical trial

## Abstract

Background: Alveolar preservation techniques for esthetic or functional purposes, or both, are a frequently used alternative for the treatment of post-extraction sockets, the aim of which is the regeneration of the lesion and the preservation of the alveolar bone crest. Methods: Studies published in PubMed (Medline), Web of Science, Embase, and Cochrane Library databases up to January 2024 were consulted. Inclusion criteria were established as intervention studies, according to the PICOs strategy: adult subjects undergoing dental extractions (participants), with alveoli treated with bone mineral grafts and collagen membranes (intervention), compared to spontaneous healing (comparison), and observing the response to treatment in clinical and radiological measures of the alveolar bone crest (outcomes). Results: We obtained 561 results and selected 12 studies. Risk of bias was assessed using the Cochrane Risk of Bias Tool, and methodological quality was assessed using the Joanna Briggs Institute. Due to the high heterogeneity of the studies (I^2^ > 75%), a random-effects meta-analysis was used. Despite the trend, no statistical significance (*p* > 0.05) was found in the experimental groups. Conclusions: The use of bone mineral grafts in combination with resorbable collagen barriers provides greater preservation of the alveolar ridge, although more clinical studies are needed.

## 1. Introduction

**Figure 1:**
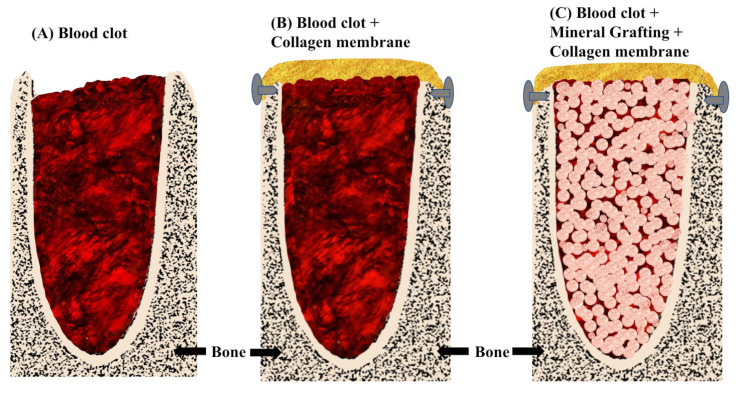
Diagram of ABRP: (**A**), Spontaneous healing; (**B**), Clot stabilization by membrane; (**C**), Bone substitute and membrane.

Dental extraction causes resorption of the alveolar ridge, starting this process immediately after extraction and causing a decrease in the vertical and horizontal dimensions of the alveolar ridge during the first 24 months [1]. To preserve the original dimensions of the ridge after extraction, either for esthetic or functional purposes or both, multiple techniques of grafting and bone substitutes in the empty socket, stabilized by resorbable or non-resorbable membranes, or simply by securing the blood clot with these membranes, have been proposed [2,3] (Figure 1).

A systematic review conducted by Avila-Ortiz et al. on 22 RCTs [4] evaluated the effect of different modalities of alveolar bone ridge preservation (ABRP) compared to spontaneous healing; however, there is currently no consensus on material choice, case selection, or appropriate clinical technique. Collagen bioresorbable membranes have been proposed as an ideal material because of their excellent tissue compatibility and low dehiscence rates, in addition to avoiding second extraction surgeries, as is the case with non-resorbable membranes [5]. Furthermore, collagen is considered a material of low immunogenicity; however, its biomedical potential is limited by its poor thermal stability and low enzymatic and mechanical resistance, so different crosslinking techniques have been proposed to minimize its degradation and improve its mechanical stability [6,7]. Based on these assumptions, a recent study by Radenković et al. concluded that the biocompatibility and stability of suitably crosslinked collagen membranes would be equal in functionality to non-resorbable membranes [8].

Graft materials need to be biocompatible and osteoconductive and provide an ideal environment for the recruitment of osteoblastic cells while being able to fill the bone defect and maintain the appropriate crestal level [9]. Although the ideal material is autologous bone, allografts, xenografts, and alloplastic materials capable of preserving the alveolar ridge have been developed and proposed in recent years. However, although these types of materials have been reported to decrease the levels of crestal resorption compared to non-grafted areas, there is no conclusive evidence to indicate the convenience of using one or another material, leaving this decision to the discretion of clinicians [10].

Alveolar healing exclusively by stabilizing the blood clot with collagen membranes has been associated with better preservation of the alveolar process compared to spontaneous healing since it has been shown that platelets can bind directly to collagen through Integrin a2b1 and Glycoprotein VI, favoring clot formation [11]. This blood clot alone induces bone maturation, differentiation, organization, and ultimately bone healing, and some studies have suggested that it may prove to be the best natural scaffold to obtain ideal bone quality and that future research on guided bone regeneration should consider the properties of the clot “per se” [12].

Nevertheless, clinicians should be aware of the possible intraoperative and postoperative complications they face when performing this type of invasive technique. The most frequent is the exposure of biomaterials, both membranes and graft materials, which, together with possible inflammation and infection, minimize the clinical results of regenerative therapy [13].

Reviews with meta-analyses focusing on the effect of collagen membranes on PRBA comparing spontaneous alveolar healing, healing by blood clot stabilization with collagen membranes, and healing with bone substitutes and collagen membranes are very scarce and, generally, focus on comparing spontaneous alveolar healing with healing with bone substitutes. Some only resort to the evaluation of these materials in what they call the “esthetic sector”. Moreover, the clinical scenarios are reduced to a single comparison: spontaneous healing and healing with bone substitutes and membranes. Our meta-analysis evaluated and compared the effect of collagen membranes on PRBA and compared spontaneous healing with healing by stabilization of the blood clot, alone or with the incorporation of bone substitutes, stabilized, in both situations, with collagen membranes.

## 2. Materials and Methods

### 2.1. Development of the Protocol and Registration

This systematic review and meta-analysis are presented in accordance with the PRISMA (Preferred Reporting Items for Systematic Reviews and Meta-Analyses) Statement and Clinical Practice Guidelines [14,15].

The protocol of our meta-analysis was registered under the INPLASY number INPLASY202420094; DOI number 10.37766/inplasy2024.2.0094.

### 2.2. Focused Interest Question

“Are collagen membranes and bone mineral grafts effective in adults, in preserving the alveolar bone crest, following tooth extraction, compared to natural alveolar healing, in terms of clinical and radiological outcomes?”

The research question was posed according to the PICOs format and included intervention studies in adult patients undergoing dental extractions (P) comparing treatment with bone mineral grafts and collagen membranes (I) with spontaneous healing of the alveolus or with other different graft materials (C) to observe the effects on clinical and radiographic parameters (O), with only randomized clinical studies (s) being considered (Table 1).

### 2.3. Data Sources and Literature Search Method

Two reviewers (NL-V, BMS) independently searched four electronic databases (MEDLINE/PubMed, Embase, Cochrane Central, Web of Science) in the last thirteen years, up to January 2024. using the terms Medical Subject Headings (MeSH): Oral Sugery Procedures* OR Surgery, Oral* AND Alveolar Bone Loss*/prevention & control AND Alveolar Process/surgery AND Alveolar Ridge Augmentation* AND Biocompatible Materials AND Bone Transplantation AND Bone Regeneration AND Guided Tissue Regeneration AND Membranes OR Collagen Membranes AND Humans*. In addition, they performed a manual search and consultations in the gray literature, as well as consultations on the bibliographic references of the studies included in the review. All this was in order to obtain as much information as possible and to avoid bibliographic bias.

### 2.4. Inclusion and Exclusion Criteria

The original research studies were selected according to the following inclusion criteria: (i) randomized clinical trials (single or double blind) that included in the study more than 10 adult subjects (≥18 years); (ii) with alveolar bone preservation needs; (iii) that provided data on clinical and radiological measurements on width, height, and volume of the alveolar bone crest; (iv) with statistical methods that included mean numerical values and standard deviation; (v) published in English. Studies that did not follow all the above criteria, with a lack of relevant data, preclinical studies or in vitro studies, case series or clinical cases, literature reviews, and irrelevant studies (letters to the Editor, congress abstracts, etc.) were excluded.

### 2.5. Assessment of the Quality of the Reports 

The included studies were methodologically evaluated using the tool developed by the Joanna Briggs Institute for RCTs (JBI MAStARI) that analyzes the methods used to synthesize the different types of evidence. The checklist consists of thirteen items whose answers are “yes”, “no”, “unclear” or “not applicable”. The answer “yes” was valued with one point. To include a study, it had to obtain a minimum score of seven [16].

### 2.6. Risk of Bias in Selected Studies

Two assessors (NL-V and BMS) independently assessed the risk of bias in the included studies using the Cochrane Risk of Bias Tool (RoB2) [17], which considers 7 domains of bias: 2 domains of selection bias (random sequence generation and Allocation concealment; 1 implementation bias (Blinding of participants and staff); 1 detection bias (blinding of outcome assessment); and 1 attrition bias (incomplete outcome data). Studies were assessed with “high,” “low,” and “borderline” risk of bias; “borderline” bias applied to that lacking information on a given bias. Discrepancies between the two reviewers were resolved by discussion and, in cases of doubt, by the opinion of a third reviewer (JABR). Cohen’s kappa index (κ) [18] was used to assess inter-reviewer agreement.

### 2.7. Meta-Analysis

Data were analyzed using Review Manager software (RevMan Software. Version 5.4.1, The Cochrane Collaboration, Copenhagen, Denmark; 2020). Due to the heterogeneity of the results, a random-effects meta-analysis was performed for studies that assessed BCW and for those that assessed BCH. CBV was reported by only one study. All were based on mean difference (MD) and standard deviation (SD) for estimating continuous data and for assessing categorical data, with 95% confidence intervals (CI). Heterogeneity was considered low with I2 = 0–30%; moderate, with I2 = 40–50%; substantial with I2 = 60–75% and high with I2 ≥ 75%. The threshold for statistical significance was set at *p* < 0.05. No meta-analysis of adverse effects was performed due to the scarcity of reports on the subject.

## 3. Results

**Figure 2:**
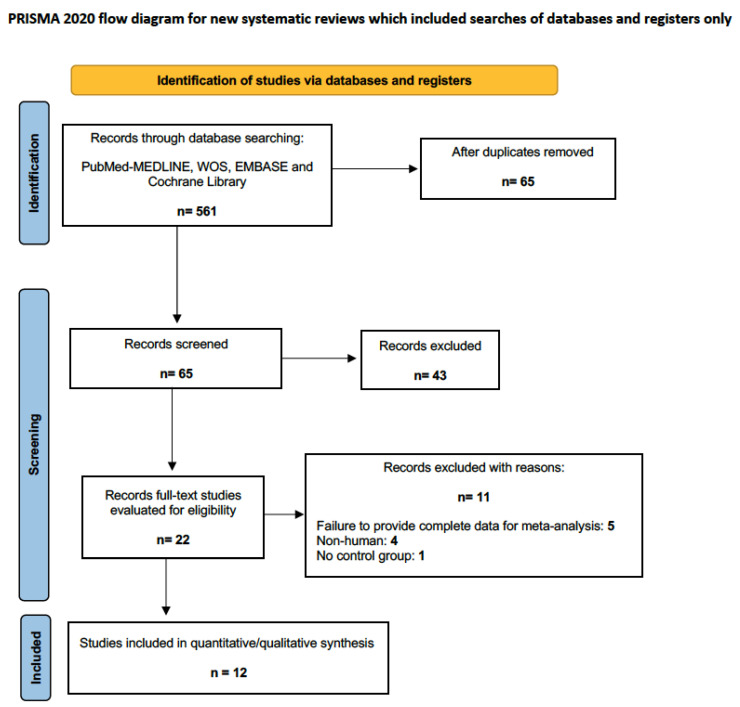
Flowchart.

The electronic search found 561 results, which, after eliminating duplicates, constituted 65 unique citations. Twenty-two full-text publications were evaluated, and 11 were excluded based on established criteria, resulting in 12 articles for evaluation [19,20,21,22,23,24,25,26,27,28,29,30] (Figure 2).

### 3.1. Study Characteristics

The included studies involved 390 adult subjects (>18 years), and 374 postextraction sockets were assessed in the different studies. Only one of the studies [23] evaluated CBV by panoramic radiograph and computed tomography, comparing alveolar filling using a bovine deproteinized bone graft coated with an absorbable collagen membrane with spontaneous healing. The number of patients ranged from 23 to 44. The study by Perelman-Karmon et al. [21] included the smallest number of subjects, and the study by Cook et al. [22] had the largest sample size. The study by Stumbras et al. [29] had the longest-term follow-up. Five studies used the molar area [23,25,26,27,28], and the others used the anterior areas of both the maxilla and mandible. The most commonly used graft material was bovine bone, together with resorbable collagen barrier membranes. Three studies used double barriers [25,26,27]. Four studies [19,20,24,25] used DBBM as a control; one, xenograft sponge [22], and six studies [23,26,27,28,29,30] used spontaneous healing as a control group. The description of the included studies is summarized in Table 2.

### 3.2. Methodological Rigor of the Studies

The methodological quality of all included studies ranged from acceptable (8 points) to very high (>10 points), as determined by the JBI-MAStARI critical appraisal checklist for RCTs. The studies that achieved the highest methodological quality were those of Jung et al., Lim et al., Cha et al. and Stumbras et al. with 12 points [26,27,28,29] and Gabay et al. [30] with 13 points (Table 3).

### 3.3. Risk of Bias within Studies

**Figure 3:**
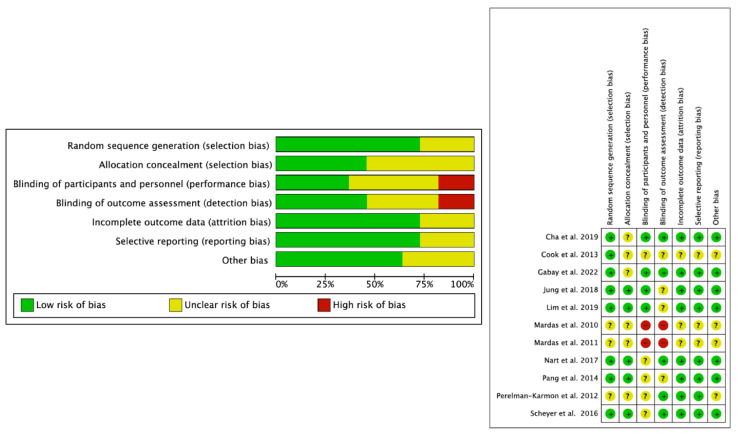
Risk of bias of meta-analysis [19,20,21,22,23,24,25,26,27,28,30].

The risk of bias assessment of included studies using the Cochrane Risk of Bias Tool (RoB2) found no studies scoring positively in all domains. Random sequence generation (selection bias), incomplete outcome data (attrition bias), and selective reporting (reporting bias) were the domains with the highest compliance. Blinding of participants and staff and blinding in outcome assessment (performance and dropout biases, respectively) were unclear or noncompliant in the included studies. Nevertheless, seven studies [24,25,26,27,28,30] met most of the domains (Figure 3).

### 3.4. Meta-Analysis

**Figure 4:**
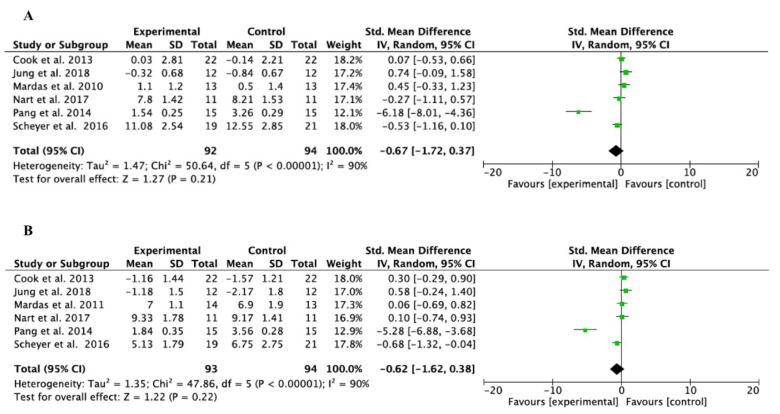
(**A**,**B**) Forest Plot of BCH and BCW (pooled studies) [20,22,23,24,25,26].

**Figure 5:**
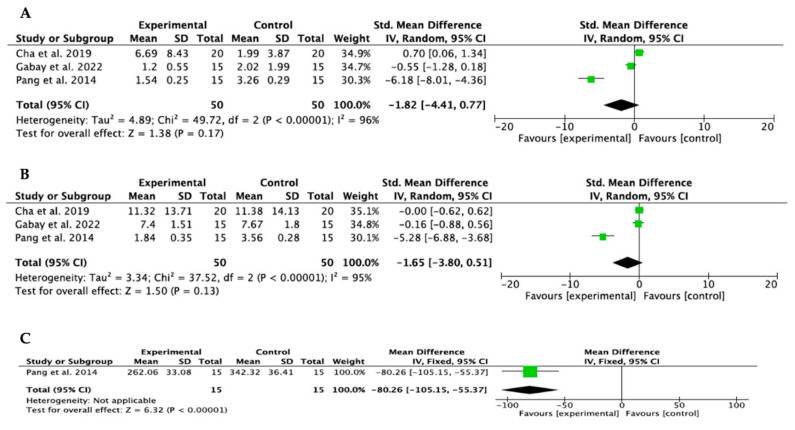
(**A**,**B**) Forest Plot of BCH and BCW from studies comparing bone mineral graft and collagen membranes with spontaneous alveolar healing [23,28,30], (**C**) Forest plot of BCW [23].

Meta-analyses were performed for pooled studies evaluating BCH and BCW (Figure 4A,B, respectively) and for those comparing bone mineral graft and collagen membranes with spontaneous healing of the postextraction alveolus (Figure 5C,D, respectively). Only the study by Pang et al. [23] compared BCW between sockets filled with bone substitutes and collagen membranes, and those that healed by spontaneous healing (Figure 5E). Heterogeneity was high in all studies (I^2^ > 75%). All studies analyzed bone mineral grafting together with collagen membranes and compared it with spontaneous healing of the alveolus. The study by Stumbras et al. [29] was not included in the meta-analysis due to a lack of data supply. The results of the meta-analyses showed a trend toward dimensional change in the bone crest in both height and width, with bone mineral grafts and collagen membranes, although without statistical significance. *p* = 0.21 and *p* = 0.22 for the BCH and BCW evaluations, respectively, in the pooled study analysis; *p* = 0.17 and *p* = 0.13 for BCH and BCW assessments, respectively, in the analysis of studies comparing bone mineral grafting and collagen membranes with spontaneous alveolar healing. Only one study assessed BCW, and, despite finding a trend toward statistical significance, it was not measurable in the meta-analysis. The studies with the greatest statistical weight were those of Cha et al. and Gabay et al. [28,30]. The Forest Plot diagrams are shown in Figure 4 and Figure 5.

### 3.5. Publication Bias

**Figure 6:**
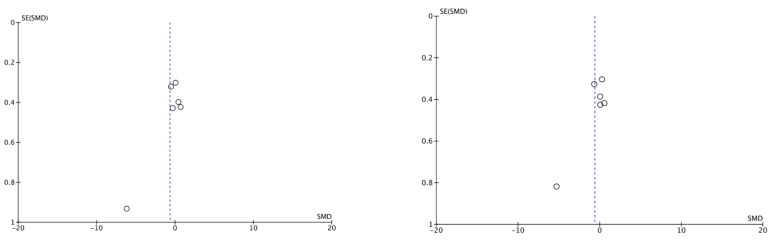
Funnel plots.

The abscissa axis (X) represents the magnitude of the effect, and the ordinate axis (Y) represents the risk effect. The middle line represents the central estimator. The symmetry of most studies demonstrates a low publication bias, although the paucity of studies limits this consideration (Figure 6).

## 4. Discussion

Since the systematic review and meta-analysis by Avila-Ortiz et al. in 2018 [4], there have been few meta-analyses evaluating ABRP using bone substitutes and resorbable membranes. Therefore, the present review and meta-analysis aim to evaluate clinically and radiographically the efficacy of bone mineral grafts and collagen membranes in ABRP after tooth extraction compared to spontaneous healing. However, we must point out that there is a lack of well-designed RCTs in the scientific literature that adequately evaluate the different regenerative techniques, and most of the studies included in our meta-analysis were parallel-arm studies, and only one was a multicenter study [25]. On the other hand, our bibliographic search was limited to the last 13 years since previous studies could be obsolete in terms of techniques and biomaterials, given the constant updating in both aspects [31].

The main methodological weaknesses observed were related to the blinding of participants and personnel, although this performance bias is difficult to overcome in RCTs of surgical interventions. Interestingly, only one of the included studies [30] was enrolled in the ClinicalTrials.gov clinical trials registry, so this study was the best evaluated in terms of methodological quality (Table 3, Figure 3).

Nevertheless, qualitative analyses showed that bone grafts together with resorbable collagen membranes provided better results than collagen barriers alone, as stabilizers of the blood clot, or spontaneous healing of the alveolus; only the study by Gabay et al. [30] did not show statistical significance in the reduction in vertical and horizontal dimensions, due, according to the authors, to a larger than expected standard deviation and minor differences between the groups. Similarly, crestal width, although only measured by one study [23] and not comparable, showed a trend toward statistical significance (Figure 5E).

These data would be of clinical relevance when preserving alveolar volume, either for esthetic or functional purposes, or both, and would be consistent with other investigations [32,33].

It has been proven by histomorphometry that sockets filled with bone mineral grafts and stabilized by resorbable membranes show, after three months, lower amounts of bone tissue compared to sockets in which the clot is stabilized or those that heal spontaneously [29]; however, the neoformed bone increases with the passage of time [30,34]. Despite these observations, the literature does not clarify this bone immaturity and its clinical repercussions, especially when used to place implanted devices, as the esthetic defect may be compensated by soft tissue growth [35,36].

Our results in the different meta-analyses showed no significant differences established as ≤0.05; however, all showed a trend towards dimensional change in the ridge in height and width, suggesting the efficacy of mineral bone grafts and collagen membranes in alveolar preservation. 

These results are far from the null hypothesis and the scientific evidence available in the scientific literature to date, so more well-designed RCTs to determine the effectiveness of the usual surgical technique of filling and sealing the alveolus in ABRP are recommended and justifiable.

### Previous Literature

Numerous preclinical and clinical studies have addressed alveolar preservation to mitigate the process of bone resorption through clot stabilization procedures and placement of bone graft material within the extraction socket with or without the application of barrier membranes, and both clinical trials and animal studies appear to be trending towards the fact that socket sealing may increase alveolar wall preservation and reduce soft tissue loss [34,37,38]. You et al. [39] conducted a retrospective study on 58 subjects to demonstrate the efficacy of atherocollagen (collagen with low immunogenicity and longer degradation time) compared to deproteinized bone mineral substitutes in alveolar ridge preservation and found no difference between the groups. Chappuis et al. [40] were pioneers in investigating three-dimensional alterations in facial esthetic zones after tooth extraction in a prospective study on 39 subjects, concluding that thin-walled phenotypes showed pronounced vertical bone resorption compared to thick-walled phenotypes. Barone et al. [34] in a large sample of 58 patients reported that alveolar filling with collagenized porcine bone and a resorbable membrane was able to maintain the width and height of the grafted areas in better condition than the spontaneously healing control areas. Subsequently, a study by Karaka et al. [41] in a small sample of patients evaluated and compared the dimensional changes in spontaneously healed postextraction sockets with those that stabilized the clot using free gingival grafts, finding no differences between the groups in terms of width but did find differences in crestal height. An extensive review by Jambhekar et al. [42] showed that, after 12 weeks of waiting, the use of grafts results in less loss of alveolar dimensions compared with ungrafted sockets. In this regard, two reviews have been performed by the Cochrane Oral Health Group, one in 2015 and another in 2021; the latter included 524 extraction sites in 426 adult participants, concluding that, although ABRP techniques can minimize changes in residual alveolar ridge height and width, after 6 months after extraction, the evidence is uncertain and that there are no significant clinical differences in terms of different grafts and occlusal barriers, recommending well-designed RCTs [43]. All this is in agreement with the results of our meta-analysis.

As for preclinical studies, a very recent one by Han et al. [44] in a canine experimental model, in which they performed volumetric analysis with computerized microtomography and histological analysis, revealed incomplete resorption, after 24 months, of bone substitutes, which would raise the need for a debate on whether or not to use such biomaterials.

Other meta-analyses, published prior to ours, assessed different modalities of alveolar ridge preservation after extraction (alveolar filling with biomaterials, collagen sponge, or autologous blood-derived products) in molar and nonmolar teeth, compared with spontaneous healing. Avila-Ortiz et al. in 2014 conducted a systematic review and meta-analysis to demonstrate the effect of alveolar filling in nonmolar teeth compared with spontaneous healing [45]. In 2020, Avila-Ortiz et al. published another review and meta-analysis that assessed alveolar ridge preservation in two clinical scenarios: (a) extraction sites with intact bone walls and (b) sites with defects such as dehiscence or fenestrations, with biomaterial grafting and barrier application, compared to spontaneous healing of the alveolus [4]. The first meta-analysis showed that alveolar fillings could significantly hinder alveolar bone remodeling after extraction due to the influence of unknown local and systemic factors, according to the authors. In the second, larger review, the analysis of pooled studies revealed that the beneficial effects of this treatment appear to be most evident only in the prevention of horizontal bone resorption. Another review with meta-analysis by Bassir et al. [46], concurring with us, compared the efficacy of alveolar ridge preservation procedures with tooth extraction without intervention and found that, although alveolar ridge preservation procedures are effective in minimizing postextraction hard tissue dimensional loss, the use of alloplastic materials produces fewer desirable results. Apaza-Bedoya et al. [47] published a recent review with meta-analysis that aimed to identify the clinical, radiographic, and histologic outcomes of alveolar ridge preservation, using bone xenografts and absorbable barriers compared to spontaneous healing in the “esthetic zone”, highlighting the efficacy of techniques and materials in reducing post-extraction crestal bone changes in this area.

Our meta-analysis evaluated and compared three different clinical scenarios: (a) spontaneous healing, (b) clot stabilization using resorbable barriers, and (c) alveolar filling with bone substitutes and resorbable sealing barriers, both in esthetic and molar areas. (Figure 1). In this regard, it would be appropriate to highlight the relevance of the blood clot even as a scaffold in bone regeneration [13,48], highlighting that some studies have proposed spontaneous healing, without using filling materials or covering the alveolar entry, stabilizing exclusively the buccal contour [49]. Aravena et al. [50] in a randomized clinical trial of split mouth in a sample of 16 subjects, found the same clinical behavior, in dimensional and volumetric terms, between spontaneous healing by blood clot and alveolar filling using autologous platelet-rich fibrin. Already in the last century, bone regeneration of a defect exclusively by clot stabilization was highlighted [51,52] and, recently, Milillo et al. [13] pointed out that the clot, on its own, stabilized with an occlusive barrier, would be able to generate a higher quality bone, although they propose the association of the clot with a filler as the main support of the clot-scaffold.

Associated with the discrepancies in research are the costs of the treatments, which are proportional to the quantity and variety of materials used. Barootchi et al. [53] recently demonstrated the economic cost-effectiveness of the different therapeutic modalities according to the results obtained, concluding that greater expenditure does not imply greater efficacy in alveolar preservation, observing only a relative association between the expenditure derived from the use of barrier membranes and alveolar preservation.

Finally, we would like to highlight a series of situations that limit this meta-analysis, which are described below: the different commercial materials used in the different studies, each with its own corresponding manufacturing characteristics; the different follow-up periods and the lack of long-term reports; and the different surgical scenarios considered, whether for esthetic or surgical-implantological purposes. All this generates clinical and methodological heterogeneity, biasing the final results.

## 5. Conclusions

Based on the results obtained in this systematic review with meta-analysis, we draw the following conclusions for clinical practice:

- The results of subgroup analyses demonstrated that ABRP using bone mineral grafts in combination with resorbable collagen barriers manifests a tendency for greater alveolar ridge preservation, both in height and width, than spontaneous healing.
- The CBV, although assessed in only one study, showed the highest tendency to statistical significance, although it could not be evaluated in the meta-analysis.
- Clinical practice can be focused in a certain direction, according to the characteristics and specific situation of each clinical case.

More well-designed clinical studies with long-term follow-up are warranted, recommended, and necessary to clarify our results.

## Figures and Tables

**Table 1:** PICOs format.

Population	Adult Subjects Undergoing Dental Extractions
Intervention	Bone mineral grafts and Collagen membranes
Comparisons	Spontaneous healing or other grafting materials
Outcomes	To observe the effects of treatment on the clinical and radiological measurements. (Δ BCW; Δ BCH; Δ CBV)
Study design	RCTs

BCW, Bone Crest Width; BCH, Bone Crest Height; CBV, Crestal Bone Volume; RCTs, Randomized Controlled Trials.

**Table 2:** Description and characteristics of the studies included.

Study, Year	Type of Study	Subjects	Experimental Area	Grafted Material	Barrier Products	Comparison	Follow-Up	Dropouts	Outcomes
Mardas et al., 2010 [19]	RCCT	30	Incisor, canine and premolar area (Maxilla and mandible)	BBM	Collagen	DBBM	8 months	4	The use of bone mineral grafts and collagen barrier in the alveolus preserved crestal height, but no differences in buccal and palatal bone plate width were observed between the groups.
Mardas et al., 2011 [20]	RCCT	30	Incisor, canine and premolar area (Maxilla and mandible)	BBM	Collagen	DBBM	4 and 8 months	3	Preservation of the alveolar ridge with bovine xenograft or synthetic bone substitute and collagen barrier also preserves radiographic bone levels.
Perelman-Karmon et al., 2012 [21]	RCT	23	Incisors and premolar area (Maxilla and mandible)	BBM	Collagen	BBM without resorbable membrane coverage.	9 months	0	The biomaterial grafted in fresh extraction sockets, together with resorbable collagen membranes, maintained its volume and favored the formation of new bone for future implant placement.
Cook et al., 2013 [22]	RCCT	44	Non-molar area	BBM	Non-cross-linked and cross-linked porcine collagen types I and III	Xenograft sponge (70% cross-linked bovine collagen type I)	21 weeks	6	The use of xenograft material along with a resorbable collagen membrane for ridge preservation in non-molar extraction sites produced significantly more vital bone.
Pang et al., 2014 [23]	RCT	30	Molar area	BBM	Collagen	Spontaneous healing	3 and 6 months	0	Bovine bone grafting together with absorbable collagen membrane were effective in preserving the alveolar ridge; however, the neoformed bone in the experimental group was of poor quality.
Scheyer et al., 2016 [24]	RCMCT	40	Molar	BBM	Collagen	DBBM with native collagen	6 months	3	The horizontal measurements of the extraction socket were significantly higher for those treated with BBM y bilayer collagen membrane. Vertical bone changes were not significant between the two treatment modalities. At 6 months, 37 of the 40 sites assessed had sufficient ridge dimension for implant placement. sufficient for implant placement
Nart et al., 2017 [25]	RCCT	26	Non-molar area	BBM	Bilayer collagen	DBBM with 10% collagen	5 months	5	A significant reduction in height and width was observed at the end of healing, but no statistically significant differences were observed between the BBM and DBBM-Collagen groups. Neoformed bone was similar in the experimental and control groups.
Jung et al., 2018 [26]	RCCT	24	Posterior areas (upper and lower jaw)	BBM	Bilayer collagen	Spontaneous healing	3 and 6 months	6	At 3 and 6 months crestal height and width were significantly higher in the test group vs. control group.
Lim et al., 2019 [27]	RCCT	33	Molar areas	BBM	Bilayer collagen and native bilayer collagen	Spontaneous healing	4 months	4	Despite the small sample size (29 sites), preservation of the horizontal alveolar ridge by open healing was advantageous; however, bone neoformation was better in the group treated with double collagen membrane, even though there was no statistical synification.
Cha et al., 2019 [28]	RCCT	40	Posterior upper molar area	BBM	Collagen	Spontaneous healing	1,3 and 6 months	1	Preservation of the alveolar ridge in the posterior maxilla using BBM grafting was more effective and resulted in less need for sinus augmentation procedures at 6 months, compared to spontaneous healing of the alveolus.
Stumbras et al., 2020 [29]	RCT	40	Anterior maxilla (Incisor, canine area)	BBM	Native collagen	Spontaneous healing	12 months	8	Significantly greater new bone formation at sites grafted with BBM and collagen membrane, compared to spontaneous healing.
Gabay et al., 2022 [30]	RCT	30	Premolar, canine or incisor area	BBM	Collagen	Spontaneous healing	6 months	2	Preservation of the alveolar ridge by DBBM resulted in a tendency to reduce the vertical and horizontal dimension.

RCCT, Randomized Controlled Clinical Trial; RCT, Randomized Clinical Trial; RCMCT, Randomized Controlled Multicenter Clinical Trial; BBM, Bovine Bone Mineral; DBBM, Deproteinized Bovine Bone Mineral.

**Table 3:** Results of the methodological assessment according to JBI MAStARI.

Study and Year	Q1	Q2	Q3	Q4	Q5	Q6	Q7	Q8	Q9	Q10	Q11	Q12	Q13	Total Score
Mardas et al. [19]	1	1	0	?	1	?	1	1	1	1	1	1	1	10
Mardas et al. [20]	1	1	0	?	1	?	1	1	1	1	1	1	1	10
Perelman-Karmon et al. [21]	?	?	1	?	?	1	1	1	1	1	1	1	?	8
Cook et al. [22]	1	1	0	?	?	?	1	1	1	1	1	1	?	8
Pang et al. [23]	1	?	1	?	?	?	1	1	1	1	1	1	1	9
Scheyer et al. [24]	1	1	1	?	1	?	1	1	1	1	1	1	1	11
Nart et al. [25]	1	1	1	?	1	?	1	1	1	1	1	1	1	11
Jung et al. [26]	1	1	1	1	1	?	1	1	1	1	1	1	1	12
Lim et al. [27]	1	1	1	1	1	?	1	1	1	1	1	1	1	12
Cha et al. [28]	1	1	1	1	1	?	1	1	1	1	1	1	1	12
Stumbras et al. [29]	1	1	1	1	1	?	1	1	1	1	1	1	1	12
Gabay et al. [30]	1	1	1	1	1	1	1	1	1	1	1	1	1	13

Q1. Was true randomization used for assigning participants to treatment groups?; Q2. Was allocation to treatment groups concealed?; Q3. Were treatment groups similar at the baseline?; Q4. Were participants blind to treatment assignment?; Q5. Were those delivering treatment blind to treatment assignment?; Q6. Were outcomes assessors blind to treatment assignment?; Q7. Were treatment groups treated identically other than the intervention of interest?; Q8. Was follow-up complete and if not, were differences between groups in terms of their follow-up adequately described and analyzed?; Q9. Were participants analyzed in the groups to which they were randomized?; Q10. Were outcomes measured in the same way for treatment groups?; Q11. Were outcomes measured in a reliable way?; Q12. Was appropriate statistical analysis used?; Q13. Was the trial design appropriate, and any deviation from the standard RCT design accounted for in the conduct and analysis of the trial?

## Data Availability

Data can be made available on request by the authors.
